# Temperature Field Numerical Analysis Mode and Verification of Quenching Heat Treatment Using Carbon Steel in Rotating Laser Scanning

**DOI:** 10.3390/ma12030534

**Published:** 2019-02-11

**Authors:** Tsung-Pin Hung, Chao-Ming Hsu, Hsiu-An Tsai, Shuo-Ching Chen, Zong-Rong Liu

**Affiliations:** 1 Department of Mechanical Engineering, Cheng Shiu University, Kaohsiung 83347, Taiwan; 2 Center for Environmental Toxin and Emerging-Contaminant Research, Cheng Shiu University, Kaohsiung 83347, Taiwan; 3 Super Micro Mass Research & Technology Center, Cheng Shiu University, Kaohsiung 83347, Taiwan; 4 Department of Mechanical Engineering, National Kaohsiung University of Science and Technology, Kaohsiung 80778, Taiwan; jammy@nkust.edu.tw; 5 Department of Industrial Upgrading Service, Metal Industries Research & Development Centre, Kaohsiung 81160, Taiwan; sat@mail.mirdc.org.tw (H.-A.T.); scchen@mail.mirdc.org.tw (S.-C.C.); zrliu@mail.mirdc.org.tw (Z.-R.L.); 6 Department of Mechanical Engineering, National Cheng Kung University, Tainan 70101, Taiwan

**Keywords:** heat treatment, rotational laser scanning, quenching, martensite

## Abstract

Temperature history and hardening depth are experimentally characterized in the rotational laser hardening process for an AISI 1045 medium carbon steel specimen. A three-dimensional finite element model is proposed to predict the temperature field distribution and hardening zone area. The laser temperature field is set up for an average distribution and scanned along a circular path. Linear motion also takes place alongside rotation. The prediction of hardening area can be increased by increasing the rotational radius, which in turn raises the processing efficiency. A good agreement is found between the experimental characterized hardness value and metallographic composition. The uniformity of the hardening area decreases with increasing laser scanning speed. The increased laser power input could help to expand the hardening depth.

## 1. Introduction

Laser surface heat treatment technology can perform surface modifications such as hardening and alloying. The composition and structure of the material surface can be changed by these methods and improve the surface hardness, wear resistance, fatigue resistance, and corrosion resistance. Compared with other traditional heat treatment techniques, such as flame heating, high frequency, carburizing, and nitriding heat treatment, laser surface heat treatment has the advantages of good strengthening effect, small thermal deformation, good processing flexibility, and automatic production, which increases the efficiency of each industry in terms of production. Therefore, the surfaces of the crankshaft, connecting rod, gear, and so forth must have high local hardness and wear-resistant parts. Most of them use steel with a carbon content of approximately 0.4% for surface hardening heat treatment. In the past decade, there has been much research on obtaining the best laser quench hardening parameters. Martínez used laser transformation hardening with scanning optics (LTHS) in the surface treatment industry and proposed a closed-loop control to maintain a nominal temperature value [1,2,3]. Different methods have been provided to control the process temperature by investigating the effect of scanning speed on the thickness variation of carbon steel’s hardened layer. A stripy spot with uniform-intensity array spots and intensity blow-up in the edge of the whole array spots were used in large-sized workpieces. Large area and uniform surface hardening treatment are required for industrial applications [4]. In addition to these requirements of the plane, the local heat treatment of complex geometric surfaces is also one of the advantages of laser hardening [5]. Barka used a 3 kW Nd:YAG laser to heat AISI 4340 cylindrical parts. The Taguchi method was used to optimize the processing parameters. In addition, for the surface hardening of large cylindrical elements, Orazi et al. proposed the use of a rotating machined part to machine the surface of a laser light ring-shaped material, which could obtain a uniform hardened layer on the surface of AISI 1040 [6]. For the choice of laser spot types, Liverani et al. assumed that the laser beam had a tophat distribution. The 2D and 3D laser heat transfer analysis modes were combined with AISI 9840 cam to verify the results. The theoretical solution can quickly and accurately predict the hardening depth and multipass processing tempering effect [7]. Tricarico et al. utilized a single-pulse laser with discrete spot irradiation of a hypereutectoid steel. By changing the defocusing distances, the laser power and pulse energy determined the influence of surface hardness and remelting area [8]. Li et al. used a high-power diode laser with a rectangular spot of uniform energy distribution, as opposed to a carbon dioxide laser, which has a circular spot with a Gaussian energy distribution. The final results showed that the surface of the workpiece was not melted under the action of the high-power diode laser and the hardness was almost the same in the workpiece. The carbon dioxide laser may cause the surface layer of the test piece to melt due to the Gaussian energy distribution. The deeper the hardened layer, the lower the hardness value [9]. Temperature is the most difficult factor to control during laser heat treatment. However, the temperature can be estimated by changing the processing parameters with numerical analysis [10,11,12,13]. In addition, the material absorptivity must also be considered in order to accurately control the temperature parameters. Skvarenina and Shin used experimental and numerical analysis to investigate the surface hardness and hardened layer depth of AISI 1536 steel under different laser quenching conditions and to estimate the absorbance of the material for the laser [14]. Kim and Lee used experimental methods to estimate the laser absorptivity of Inconel 718 nickel-based alloy and proposed a prediction equation for the cutting force and preheating temperature of laser-assisted milling [15]. The experimental equipment used in this study is the same as the laser-assisted milling system used in [16]. This equipment provides a compounding process. For example, the heat treatment can be performed after milling or simultaneously with milling by laser preheating. The current study will provide a numerical and experimental analysis of the thermal response of the AISI1045 workpiece material in the laser quenching process. The hardening zone of quenching induced by rotational laser scanning will be experimentally measured and predicted by finite element modelling. The hardening depth will be analyzed from the measured data and model prediction. A novel coaxial laser heating spindle will be proposed for the heat treatment process. The temperature field prediction induced by a rotational laser scanning path will be provided. A parametric study will be conducted to investigate the effect of laser power and feed rate on the temperature field.

## 2. Finite Element Analysis Modelling

**Figure 1:**
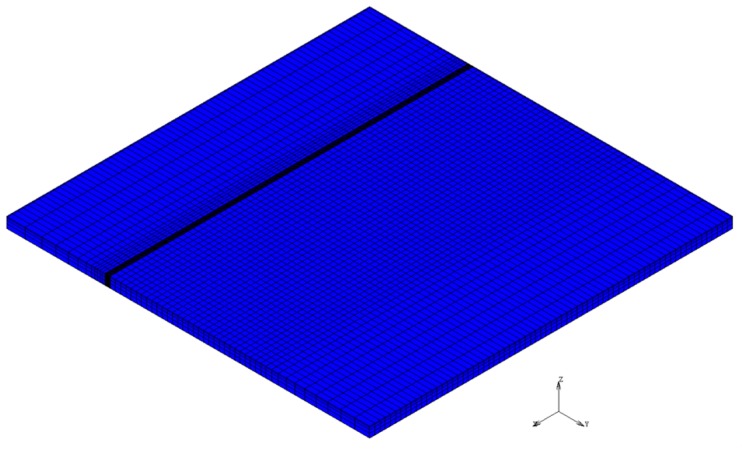
Proposed laser heat treatment finite element model.

In the previous research, the author proposed a finite element analysis model of laser Gaussian energy distribution [17], using a single-pass laser to discuss the effects of different processing parameters on the hardened zone of medium carbon steel. The numerical analyses of this study were also performed using the thermoelastic–plastic models of the commercial MSC Marc software suite for finite element analysis. Coupled thermomechanical analysis was used to improve the physical accuracy of our calculations. During each iteration of the simulation, the actual temperature distribution of the model is acquired and the corresponding strains and stresses of the model are also calculated. This ensures that the model will, at any point in time, satisfy all equilibrium equations and convergence conditions. This method of numerical analysis produces results that are more accurate than uncoupled thermomechanical methods. To ensure a reasonable level of computational efficiency, a simple 70 mm × 70 mm × 2 mm model was used. In the finite element analysis model, an eight-node hexahedral element was used. The number of elements and nodes was 17,360 and 22,365, respectively, as shown in Figure 1. The mechanical properties of AISI 1045 [18] steel is shown in Table 1. Based on the existing literature, the quenching and melting temperatures of AISI 1045 are 760 °C and 1520 °C, respectively. These temperature ranges were used to determine whether a material was successfully hardened.

On the boundary condition setting, it is assumed that the laser energy is focused on the surface of the material. The initial temperature of the material was set to 25 °C, and the temperature of the material was reduced to room temperature in consideration of the natural heat convection effect of the air, and the heat convection coefficient was assumed to be 12.6 W/m^2^·°C. 

### 2.1. Laser Heat Source Modeling

The laser power distribution on the laser beam focus plane is described in terms of an average distribution, as seen below:

(1) $$P_{e}=\eta_{e}\frac{P_{i}}{\pi R_{e}^{2}}$$

where $P_{e}$ is the energy absorbed by the material, $P_{i}$ is the laser power, $\eta_{e}$ is the absorption rate of the laser energy for the material, and $R_{e}$ is the radius of the laser spot. As the vast majority of materials cannot fully absorb the energy provided by the laser, it is necessary to account for the laser energy absorption rate of the material used; that is, $\eta_{e}$. Based on the data provided by references [13,15,19,20,21], $\eta_{e}$ was defined as 35%. 

The mathematical function for $P_{e}$ was written in the Fortran programming language. Using the subroutine “Flux” interface provided by the boundary condition function in MSC Marc, the numerical values calculated using the Fortran code were fed into MSC Marc to be used in subsequent calculations [22]. These calculations are as follows: In each time step, the Flux subroutine is called during each Gaussian integration point of the analysis, with the appropriate flux type being specified in the DIST FLUXES input option. The flux type is chosen according to the element type. The equivalent heat flux obtained from the mathematical function of $P_{e}$ at each node is then calculated and stored. 

**Figure 3:**
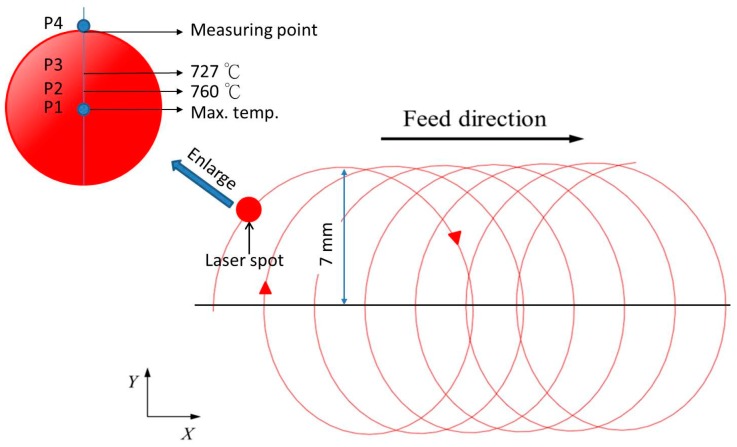
Schematic diagram of rotational laser scanning path.

In this study, a coaxial laser heating system is used for the quenching process. The first attempt to use coaxial laser heating was made by Brecher et al. [23]. The author also studied the use of coaxial lasers for preheated milling. This article uses the same equipment. Instead of traditional heat treatment to heat the whole workpiece, a local heating scheme is introduced. The partially quenched area can be created on the workpiece, maintaining the toughness inside the material. In addition to the translation in the *x*-direction, the laser rotates along with the workpiece in the milling process at a constant angular speed, as shown in Figure 3.

### 2.2. FE Model for Circular Scanning of Moving Heat Source 

**Figure 4:**
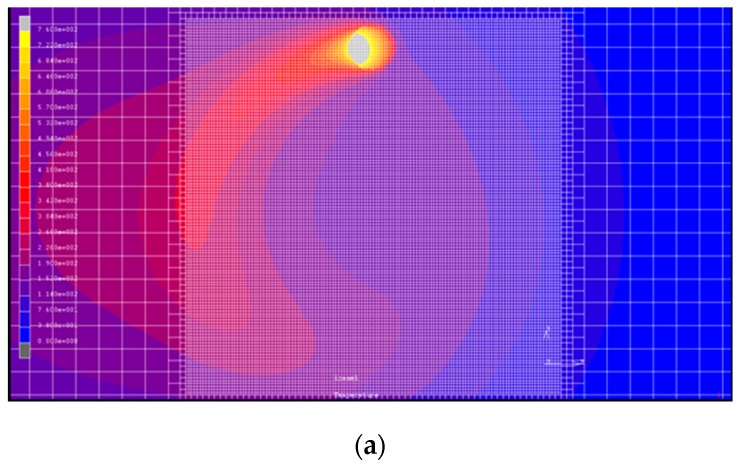

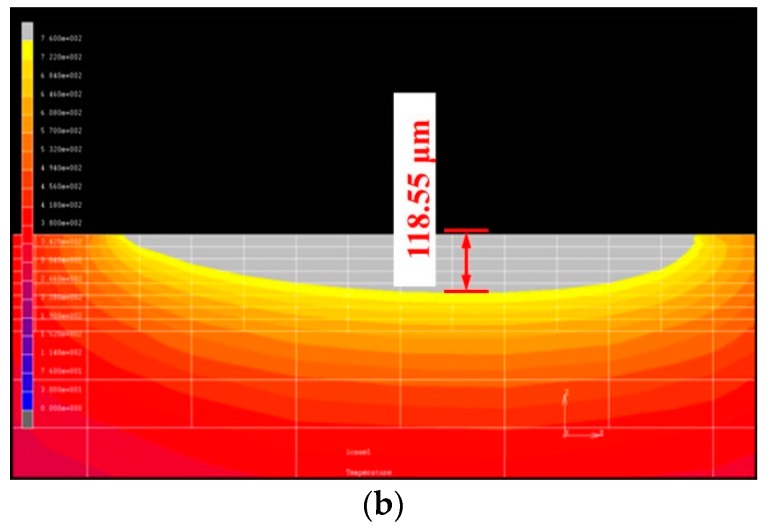
Schematic diagram of moving local grid redivision and hardening zones. Hardening zone of (**a**) top view and (**b**) cross section with laser power of 500 W, rotational speed of 100 rpm, and feed rate of 100 mm/min.

In the heat treatment of medium carbon steel, when the temperature above the A1 phase change point reaches or more [24], it cools rapidly and the hardness of the material surface increases considerably. Therefore, in this paper, the temperature distribution after finite element analysis is used to determine the area with a temperature higher than 760 °C within the hardened region. The hardened zone determination is represented by a color-matching temperature gradient, as shown in Figure 4. The temperature indicated in white, that is, over 760 °C, denotes that the temperature in this area has reached the quenching temperature.

**Figure 5:**
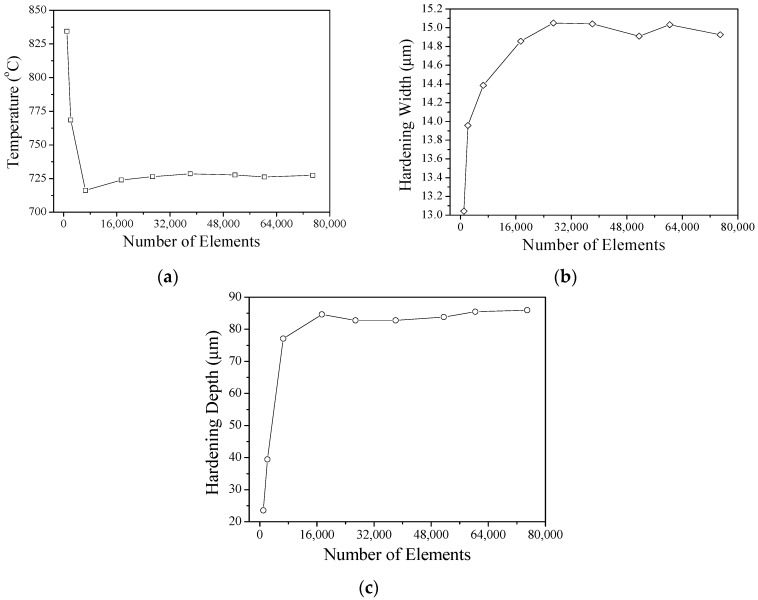
(**a**) Convergence analysis with temperature, (**b**) convergence analysis with hardening width, and (**c**) convergence analysis with hardening depth.

The internal temperature gradient of the material is extremely narrow due to the local heating of the material by the YAG laser. During the calculation, when the analysis data diverge, the mesh is partially refined. The remeshed element is used to refine the heat-affected zone range and improve data accuracy. As the number of elements cannot be re-refined indefinitely, the mesh convergence analysis used increase the efficiency of the calculation is shown in Figure 5. The results show that when the number of meshes reaches 17,360 or more, the values of the highest temperature, hardening width, and depth of the hardened layer of the material are almost stable. Therefore, going forward, the number of analyzed mesh elements should exceed 17,360.

Since the model mesh uses eight-node hexahedral elements with four Gaussian integration points on each side, the elements are proportional to the number of Gaussian integration points. Thus, the mesh density and number of Gaussian integration points at the laser focus point affect the accuracy of its heat source distribution. In order to represent the mesh density of the laser heat source distribution fineness, the overall model is divided and the remesh setting is used. The subroutine program “Uadapbox” is applied to define the area of the local mesh redivision. The number of elements in the grid redivision is set by levels. For each level, the hexahedral elements will increase by a factor of eight. All the models established in this paper use level 3 as the setting.

The redivided area of the mesh has a box-shaped boundary, and the area is set within the rotation range of the laser heat source. The boundary has the same moving speed. The mesh redivision starts when the moving boundary enters the grid. At this time, the number of elements in the area will rise sharply and the total amount of elements will increase rapidly as the boundary moves. This not only reduces the efficiency of the solution, but also uses a large number of computer resources. Therefore, a subroutine must be set in the function to ensure that the subdivided mesh returns to the initial mesh size after leaving the box boundary, as shown in Figure 4. Thus, the accuracy of the simulation can be maintained and the efficiency of the solution can be improved while saving computer resources.

## 3. Experimental Setup

**Figure 6:**
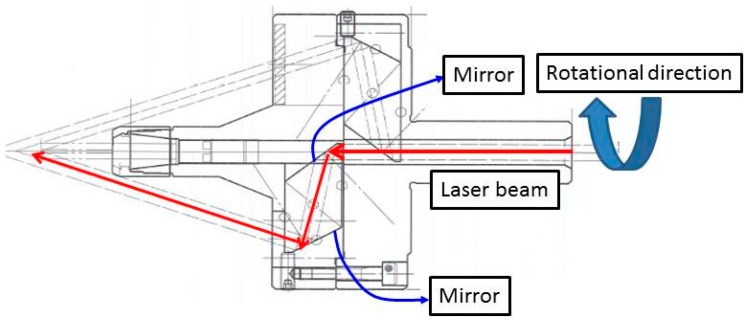
Laser beam path in the coaxial laser heating spindle.

**Figure 7:**
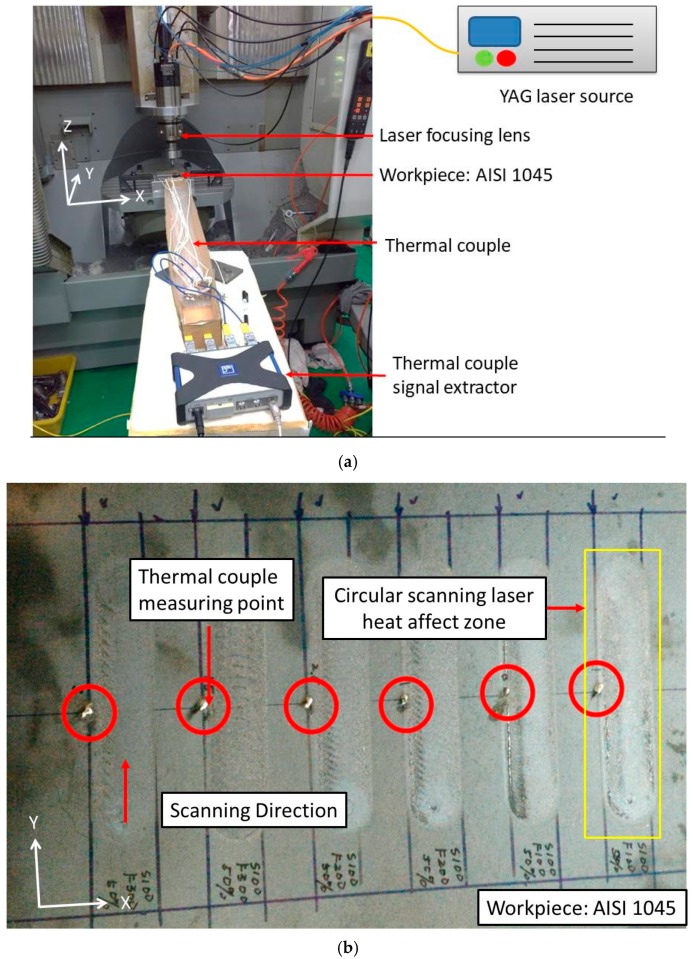
Experimental setup. (**a**) The arrangement of the laser, workpiece, and thermal couple; and (**b**) thermocouple setup on the workpiece and quenching tracks.

During the experiment, a coaxial laser heating system was used for the laser hardening process, as shown in Figure 6. The laser lens and cooling water were set beside the CNC spindle, as shown in Figure 7a. A 1064-nm YAG laser with a maximum power of 1200 W was used in the experiment. 

The dimensions of the AISI 1045 medium carbon specimen used in this experiment were 210 mm × 170 mm × 2 mm. Various laser powers were used to simplify the numerical calculations at different scanning speeds (ranging from 400 W to 900 W), and the spindle feed rate was 100 mm/s. The diameter of the laser spot was 2 mm and the laser rotational radius was 7 mm. The thermocouple was welded at a distance of 8 mm from the centre of the weld path, as shown in Figure 7b. The Vickers hardness testing instrument was used to measure the surface hardness via laser heating during the hardness measurement experiment.

## 4. Results and Discussion

### 4.1. Results of Finite Element Analysis and Experimental Results

**Figure 8:**
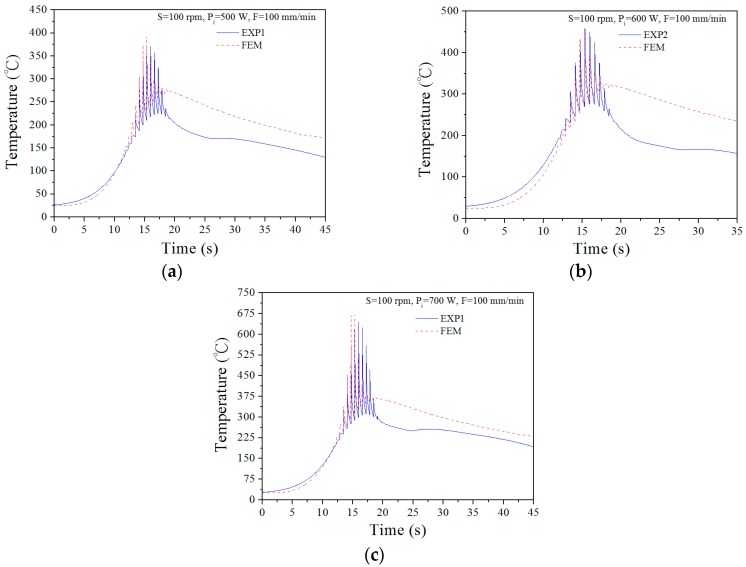
Comparison of surface temperature between the experiment and FEM modelling at a laser scanning speed of F = 100 mm/min when the laser power input is (**a**) P_i_ = 500 W, (**b**) P_i_ = 600 W, and (**c**) P_i_ = 700 W, where rotational spindle speed S = 100 rpm.

The top surface temperature history of the workpiece, which is located 0.5 mm away from the laser beam heating centre, is plotted as a function of time for different laser power inputs in Figure 8. The temperature increases sharply when the laser spot approaches the measurement point and then cools to the room temperature. When the laser power input decreases from 500 W to 700 W, the peak temperature shows a slight increase from 390 °C to 670 °C. This also means that the temperature at the centre of the laser beam will be higher than the measured value. The temperature change history shows that as the laser spot shifts closer to the measurement point, the temperature rises, and it falls as the laser moves away. Therefore, the data contain a cyclic pattern due to the temperature rise and drop. When the laser is located far away from the measuring point, the temperatures of the heating and cooling gradient are balanced. Simultaneously, the temperature will decrease steadily. The predicted temperature history profiles agree well with the measurements.

**Figure 9:**
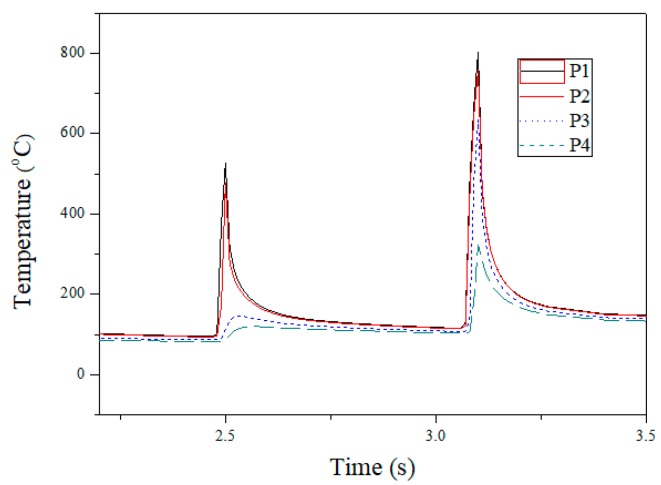
Finite element analysis of temperature history prediction in the laser beam core.

Figure 9 shows the prediction of the temperature variation within the laser spot and the experimental measurement. The reference points of the value are shown in Figure 3 (P1–P4). P1 is located at the centre of the laser spot. The laser heats the material locally above temperature A1 (approximately 727 °C) and the material changes to the austenite phase and is then air-cooled naturally. Taking experimental parameter no. 2 as an example, as shown in Table 2, the highest temperature range of P1–P2 lies between 803.9 and 760 °C. As the laser-heated area is small relative to the total area of the material, the air-cooling time is less than 1 s and the martensite phase is formed at a temperature of 200–300 °C.

According to the continuous cooling transformation curve, when the cooling critical rate is greater than 200 °C/s, the austenite phase will be transformed into the martensite phase [23]. The maximum temperature is less than 760 °C at P3 and P4, and thus, there is no quench hardening phenomenon. 

**Figure 10:**
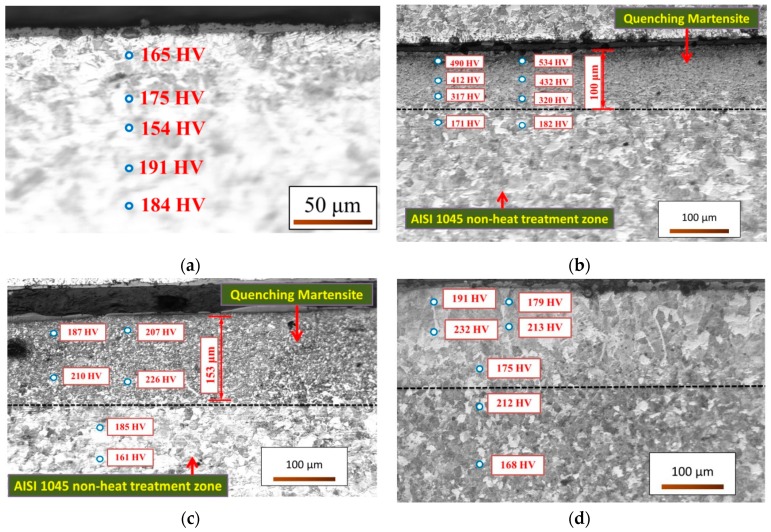
Cross-section hardness measurements of AISI 1045 steel at rotational speed of 100 rpm, feed rate of 100 mm/min, and laser power of (**a**) 400 W, (**b**) 500 W, (**c**) 600 W, and (**d**) 900 W. (Vickers hardness test)

Table 2 and Table 3 show the changes in the surface and cross-section hardness of the material after quenching by heating the material at different laser powers. It can be inferred that when the laser power is 400 W, the temperature does not reach the austenite phase transfer temperature. The metallographic structure of the cross section indicates that the surface structure is the same as the inner layer and martensite formation does not take place. When the laser power is 500 and 600 W, austenite forms. After air cooling, the surface structure becomes martensite and the hardness distribution decreases from the surface of the material to the inside. The hardening depth is 100 μm and 153 μm, respectively. When the laser power is 900 W, the temperature is too high. This causes the surface layer of the material to become molten. After cooling, the grain size is larger than the non-heat-treated area and no martensite formation occurs in this zone, as shown in Figure 10d.

**Figure 11:**
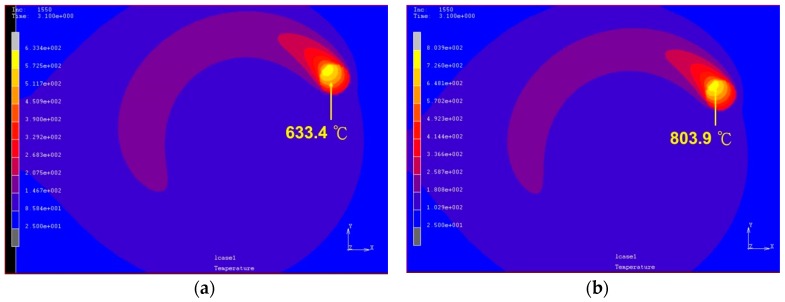

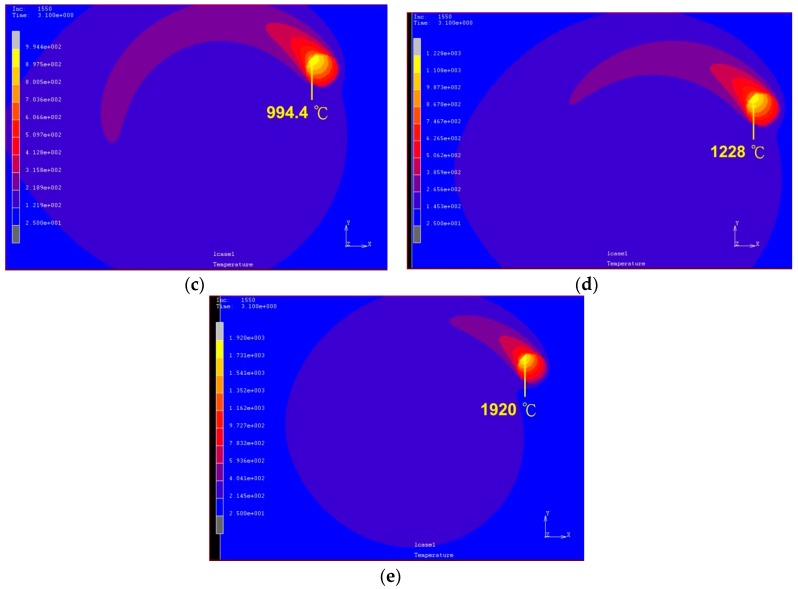
Top view of temperature field induced by a laser rotating at a rotational speed of 100 rpm, with a 7 mm radius, *x*-directional moving speed of 100 mm/min, and power of (**a**) 400 W; (**b**) 500 W; (**c**) 600 W; (**d**) 700 W; and (**e**) 900 W.

The top views of the predicted temperature fields are shown in Figure 11. The maximum predicted temperature for a laser power of 400 W is 633.4 °C, which is below the material quenching temperature. The results in Table 2 and Table 3 are compared with Figure 11a, and it is notable that the material in the heating zone does not change to the γFe phase. Moreover, there is no phase change in the structure during the cooling process. Figure 11b shows that the maximum temperature of the material is 803.9 °C and the quenching temperature has reached the heat-affected zone. The hardness tests verify that the hardness of the material can be increased by 141.79% (from 317.3 HV to 767.2 HV) under a power of 500 W, rotational speed of 100 rpm, and feed rate of 100 mm/min. When the laser power is 600 W, the maximum temperature of the heat-affected zone is estimated to be 994.4 °C and the material is δFe. The hardness after cooling increases by 14.47%. When the laser power is greater than 700 W, the material temperature is between the softening and liquefaction points. Therefore, the increase in the hardness of the material below this temperature after cooling is less than 30%. 

### 4.2. Finite Element Prediction: Parametric Study

**Figure 12:**
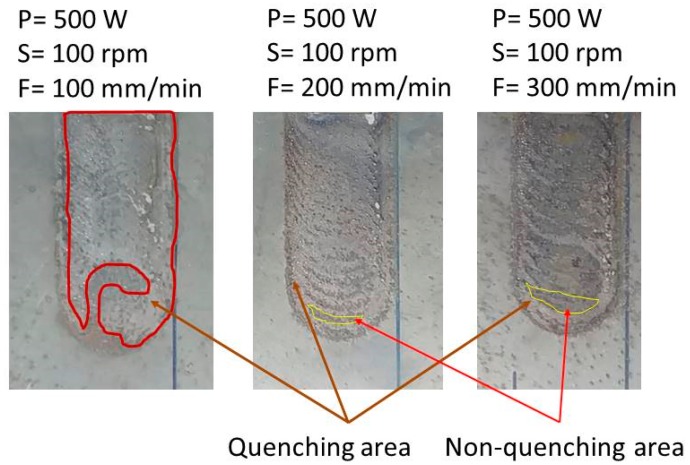
Distribution of quenching zone at different feed rates.

The comparison of the temperature prediction and experimental results at different laser powers and feed rates is shown in Table 4. The highest temperature error between the predicted data and experimental results measured at P4 is less than 7%. Under fixed laser power, as the feed rate increases, the temperature in the material heat-affected zone decreases, which will be detrimental to the quenching process. When the feed rate increases, the quenching area becomes uneven, as shown in Figure 12. The light grey area is the quenching area when the feed rate is 200–300 mm/min. Because the rotation speed cannot match the feed rate, some areas are not heated by the laser. They are highlighted by the area encircled by the yellow lines. Table 4 also shows that the predicted temperature at P1 is higher than 727 °C. This means that the laser power should be greater than 500 W as long as the feed rate is properly matched with the spindle speed. A uniform quench-hardened zone can thus be obtained.

## 5. Conclusions

The temperature distribution in rotational laser scanning for AISI 1045 steel was experimentally investigated. A three-dimensional finite element model was developed for the temperature field prediction. The predicted temperature fields showed close approximation to the experimental measurements. The hardening zone was also predicted by comparing the simulated isotherm with the experimentally characterized hardness value and metallographic composition. A parametric study was conducted to investigate the effects of laser scanning speed and power input on the hardening zone. The hardening zone uniformity decreased with increasing laser scanning speed. The increased laser power input could help expand the melting zone area with a laser power range of 500–600 W, rotational speed of 100 rpm, and feed rate of 100 mm/min. Thus, the finite element model can be applied to make temperature distribution predictions in coaxial laser heating systems.

## Figures and Tables

**Figure 2:**
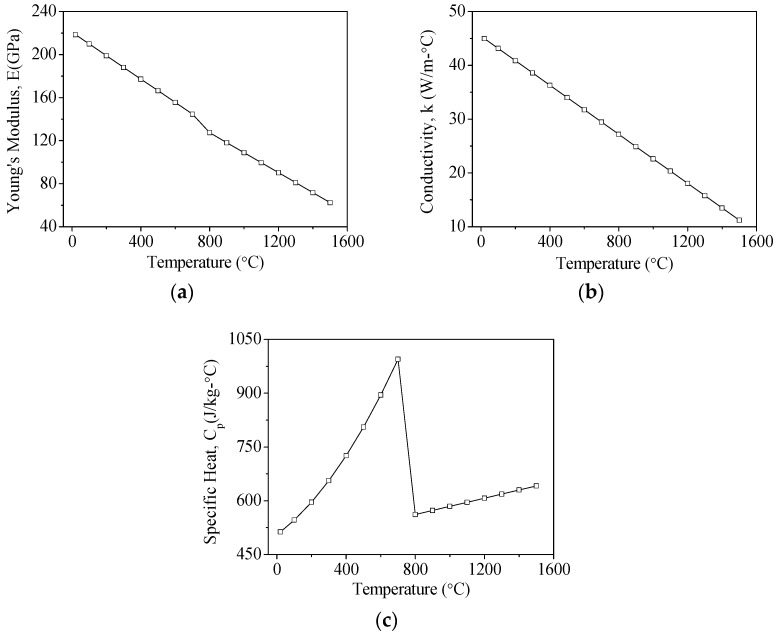
The temperature-dependent material properties of AISI 1045 steel: (**a**) Young’s modulus; (**b**) coefficient of thermal conductivity; and (**c**) specific heat.

**Table 1:** Basic material properties of AISI 1045 steel.

Property	Values
Density (kg/m^3^)	7870
Thermal Conductivity (W/m·°C)	Figure 2
Specific Heat (J/kg·°C)	Figure 2
Young’s Modulus (GPa)	Figure 2
Yield Strength (MPa)	310
Coefficient of Thermal Expansion (CTE, μm/m·°C)	15
Poisson’s Ratio	0.27
Hardening Temperature $T_{h}$ (°C)	760
Melting Temperature $T_{m}$ (°C)	1520
Tempering Temperature $T_{t}$ (°C)	400

**Table 2:** Surface hardness measurements of AISI 1045 steel.

S = 100 rpm, F = 100 mm/min				
EXP. No.	Laser Power (W)	Non-Heat-Treatment Hardness (HV)	After Quenching Hardness (HV)	Hardness Increase or Decrease Rate (%)
1	400	317.3	222.2	−29.97
2	500		767.2	141.79
3	600		363.2	14.47
4	900		407.9	28.55

**Table 3:** Cross-section hardness measurements of AISI 1045 steel.

S = 100 rpm, F = 100 mm/min					
EXP. No.	Laser Power (W)	Experimental Hardness Depth (μm)	Min. Hardness at Cross Section (HV)	Max. Hardness at Cross Section (HV)	FEM Hardness Depth (μm)
1	400	0	165	191	0
2	500	100	171	534	118.55
3	600	153	161	226	181.38
4	900	0	168	232	0

**Table 4:** Maximum temperature prediction and verification at the measuring point.

Laser Power (W)	Feed Rate (mm/min)	FEM Max. Temperature (°C) (at P1)	EXP. Max. Temperature (°C) (at P4)	FEM Max. Temperature (°C) (at P4)	Prediction Error (%) (at P4)
500	100	803.9	370.8	392.29	5.8
	200	774.4	164.9	165.49	0.36
	300	758.5	85.1	90.49	6.33
600	100	994.4	298.3	292.52	−1.94
	200	922.1	254.1	262.84	3.44
	300	898.9	186.4	182.35	−2.17
700	100	1228	352.5	354.84	0.66
	200	1118	341.3	344.64	0.98
	300	1082	225.8	223.44	−1.05
